# Squamous Papilloma of the Esophagus in a Patient With Refractory Gastroesophageal Reflux Disease

**DOI:** 10.7759/cureus.87691

**Published:** 2025-07-10

**Authors:** Shivam Gandhi, Wesam Frandah, John Diks

**Affiliations:** 1 Gastroenterology, Marshall University Joan C. Edwards School of Medicine, Huntington, USA; 2 Internal Medicine/Gastroenterology, Marshall University Joan C. Edwards School of Medicine, Huntington, USA; 3 Pathology, Marshall University Joan C. Edwards School of Medicine, Huntington, USA

**Keywords:** endoscopy, esophagus, gastroesophageal reflux disease, refractory gerd, squamous papilloma

## Abstract

We report a case of a 35-year-old woman with persistent gastroesophageal reflux disease (GERD) despite lifestyle modification and medical therapy. Endoscopy revealed a squamous papilloma in the distal esophagus, a rare benign lesion linked to chronic mucosal irritation. The lesion was excised, and histopathology confirmed squamous papilloma without dysplasia. This case underscores the importance of endoscopic evaluation in patients with uncontrolled reflux and highlights the potential link between GERD and benign esophageal neoplasms.

## Introduction

Esophageal squamous papilloma (ESP) is a rare, benign tumor with an estimated prevalence of 0.01% to 0.45% in endoscopic series, with an unclear cause and pathogenesis [1-4]. ESP is exceedingly rare, comprising only a minute fraction of esophageal lesions with fewer than 200 cases reported by the mid-2010s, although a 2016 case-control study found a prevalence of 0.72% in an endoscopy population [2,3]. There is no consistent strong gender predilection across all studies; some series from Western countries have reported a slight male predominance or roughly equal sex distribution [3], whereas certain cohorts have found an African American predominance. Patients are usually middle-aged at diagnosis (median age around 50 years) [3,5]. 

The Paris endoscopic classification is a standardized system established to categorize superficial neoplastic lesions in the esophagus, stomach, and colon based on their morphology. It outlines lesion types (e.g., protruding, flat, depressed) to improve diagnostic accuracy, guide therapeutic decisions, and ensure consistency across studies. Although the Paris classification is not explicitly referenced in all ESP literature, endoscopically, these lesions often appear as small, protruding nodules and are morphologically consistent with Paris type Is (sessile) lesions. They may appear as whitish or pink, exophytic, wart-like projections with a lobulated or smooth surface [6-10]. The differential diagnosis includes verrucous squamous cell carcinoma, glycogenic acanthosis, and inflammatory polyps, making histologic confirmation essential [6,10]. 

ESPs likely arise through a combination of chronic mucosal injury, inflammatory stimulus, and (in some cases) oncogenic viral infection or genetic factors, leading to a benign papillary proliferation of the esophageal squamous epithelium [3-5,7,8]. 

The exact etiology and pathogenesis of ESP remain incompletely understood [4]. Chronic mucosal irritation, for example, repeated chemical injury from gastroesophageal reflux disease (GERD) or mechanical trauma from interventions like dilations and stents, is thought to precipitate these lesions by inducing cycles of epithelial damage and regenerative hyperplasia [3,7,8]. Consistent with this, associations have been noted with reflux esophagitis, Barrett’s esophagus, and exposure to irritants such as tobacco and alcohol, supporting a role for inflammation-driven pathogenesis [5,8]. Human papillomavirus (HPV) infection has also been implicated: HPV DNA is detectable in a subset of esophageal papillomas (approximately 20% in aggregate analyses), although reported detection rates vary widely across studies [3,4]. Low-risk HPV genotypes (e.g., HPV-6 and HPV-11) are the most frequently identified in these tumors, with occasional high-risk subtypes like HPV-16 reported, but many papillomas are HPV-negative, especially in recent series, indicating that viral infection is not a universal cause [3,5]. Other infections (such as Epstein-Barr virus) have been sporadically proposed, and rare genetic predispositions have been described [5]. Some cases of diffuse esophageal papillomatosis occur in the context of inherited syndromes like focal dermal hypoplasia (Goltz syndrome) or Cowden syndrome, suggesting a contributory role of genetic mutations in susceptible individuals [5,8]. 

Most patients with ESP are asymptomatic, and lesions are often discovered incidentally during endoscopy performed for unrelated indications [5,6]. When symptoms do occur, they are typically nonspecific and may include mild dysphagia, throat discomfort, or vague chest symptoms, often attributed to coexisting conditions like GERD [5,6]. Management of ESP centers on complete endoscopic removal, usually performed at the time of biopsy or polypectomy [3,8]. This approach is both diagnostic and therapeutic, as histological examination is required to differentiate ESP from other papillary or neoplastic lesions [6,10]. The prognosis is excellent following complete excision, with recurrence being rare and most patients remaining symptom-free [8]. Although malignant transformation is exceptionally rare, it has been reported, particularly in the context of multiple lesions or high-risk histologic features, prompting some authors to recommend follow-up endoscopy in selected cases [3,5,10]. Overall, ESP is a benign condition with favorable outcomes in the vast majority of cases when appropriately managed [8,10]. 

GERD is a common chronic condition characterized by the reflux of gastric contents into the esophagus, leading to symptoms such as heartburn and regurgitation, with a reported prevalence of up to 20% in Western populations [11]. Diagnosis relies on a combination of clinical presentation and objective testing, including upper endoscopy, esophageal pH monitoring, manometry, and impedance testing [12]. Refractory GERD is defined by the persistence of typical symptoms despite at least eight weeks of optimal proton pump inhibitor therapy and may require further evaluation to rule out functional disorders or alternative diagnoses [13]. While the pathogenesis of ESP remains unclear, chronic mucosal irritation from GERD has been proposed as a contributing factor, particularly in lesions located in the distal esophagus [1,4]. However, the evidence for this association remains limited and inconclusive.

## Case presentation

A 35-year-old woman presented with a four-year history of chest tightness and nocturnal gastroesophageal reflux unresponsive to acid suppression therapy. She was a former smoker and quit 15 years ago. Initially diagnosed with anxiety and treated with clonazepam, her symptoms improved partially. Suspecting GERD, her primary physician prescribed pantoprazole, which provided symptomatic relief. She discontinued it due to concerns about long-term use and attempted dietary modification and weight loss, but experienced rebound symptoms. She was switched to esomeprazole (Nexium), with some improvement, but she continued to experience nocturnal reflux with coughing and chest tightness, which persisted for over eight weeks. She denied dysphagia, hematemesis, melena, weight loss, vomiting, or fever. She had no prior abdominal surgeries and denied NSAID use. 

**Figure 1:**
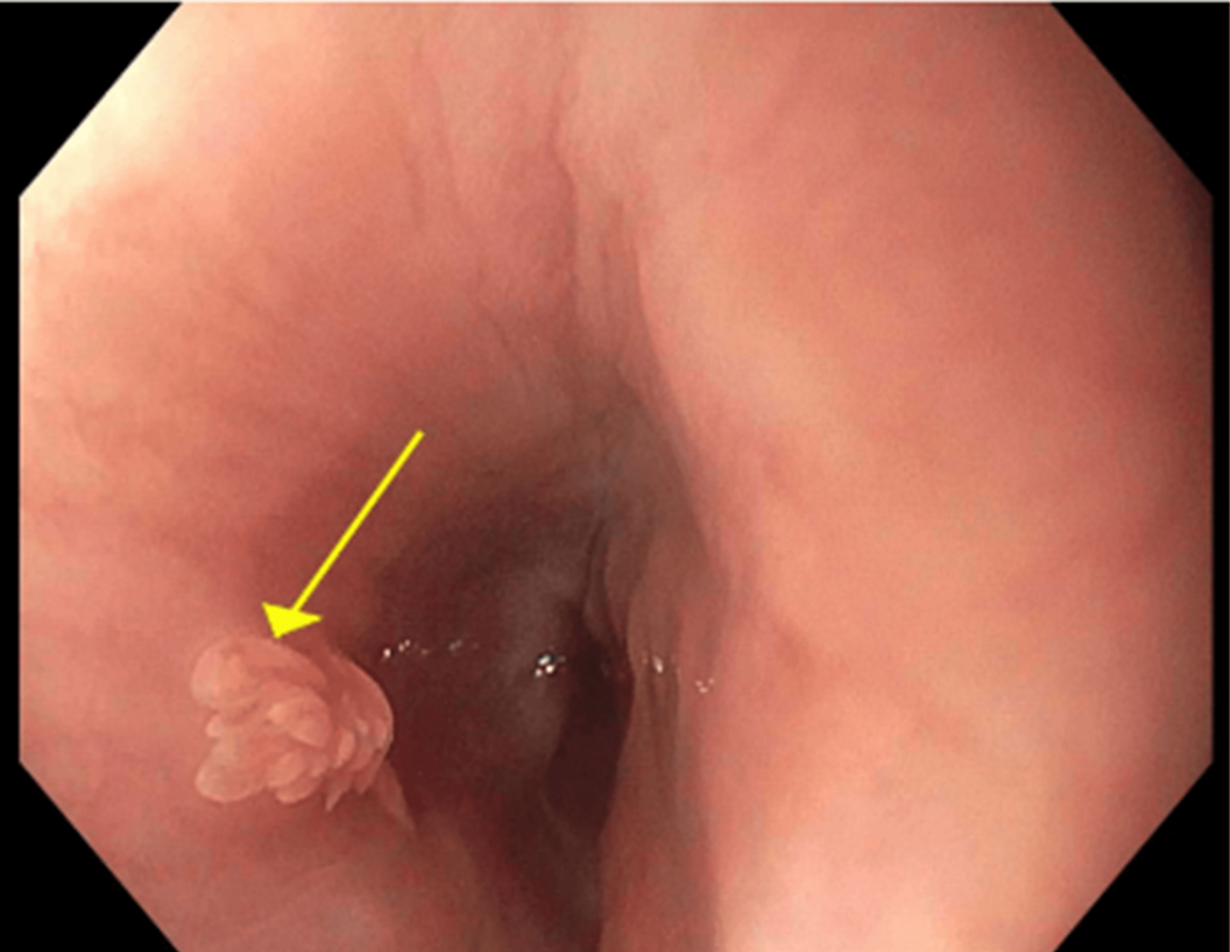
Endoscopic view of the sessile esophageal nodule, showing a lobulated, wart-like surface and broad base consistent with a non-pedunculated protruding lesion (yellow arrow). High-resolution image captured prior to resection with cold biopsy forceps.

Esophagogastroduodenoscopy revealed a sessile 5 mm lesion with a lobulated surface and broad base, consistent with a non-pedunculated protruding lesion in the distal esophagus approximately 32 cm from incisors (Figure 1). The lesion was removed during the procedure. Functional lumen imaging probe (FLIP) studies showed normal esophageal contractility and a normal esophagogastric junction opening. A BRAVO pH capsule was successfully deployed for ambulatory acid monitoring, which revealed an overall controlled acid reflux and DeMeester score of 6.7 (normal ≤14.72, 95th percentile). The stomach and duodenum appeared normal and were biopsied.

**Figure 2:**
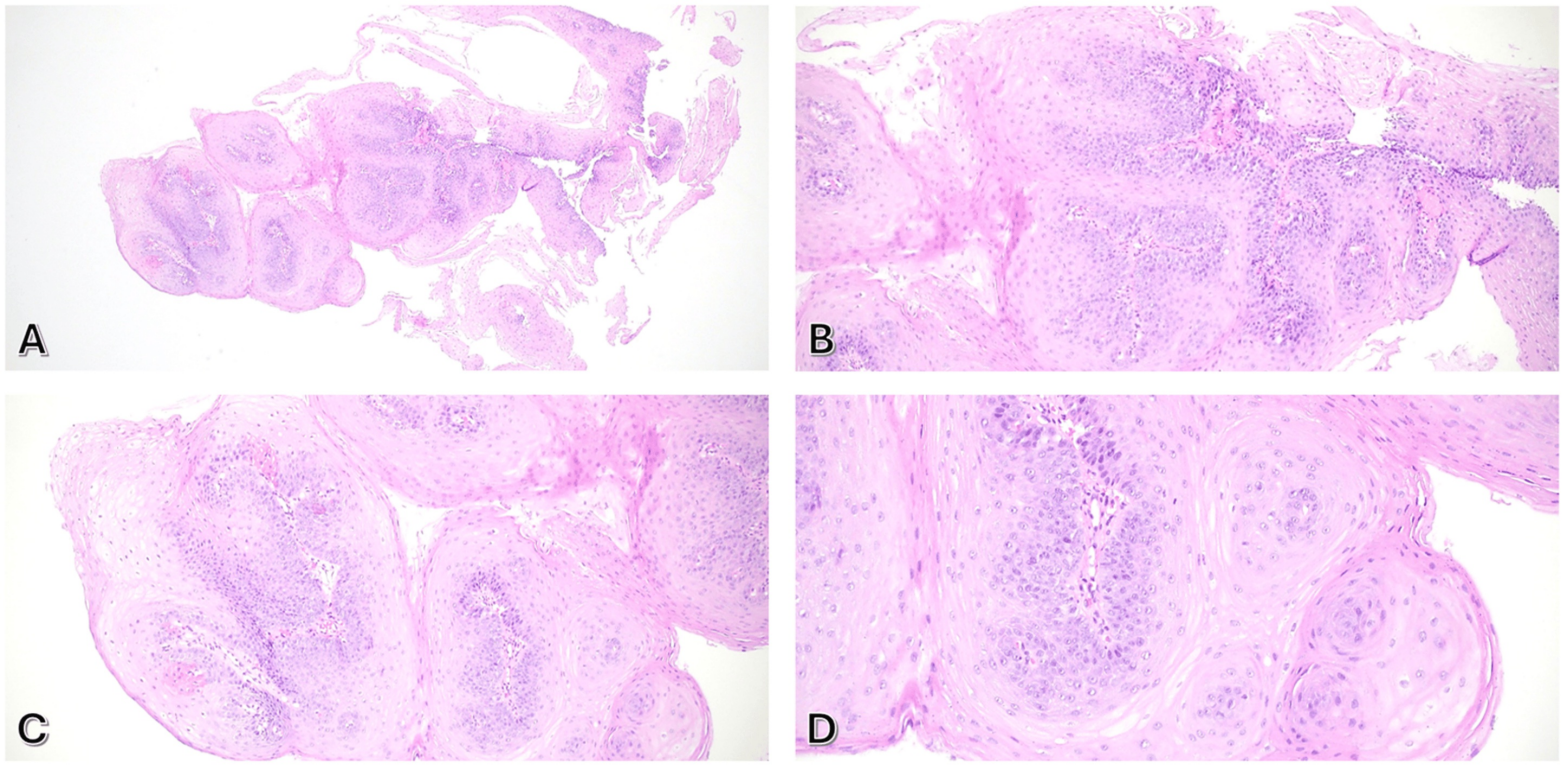
Histological images showed papillary fronds with mature squamous epithelium and fibrovascular cores, with no dysplasia or malignancy observed. Hematoxylin and Eosin (H&E) images (A: x40, B&C: x100, D: x200).

Histological analysis of the esophageal nodule showed papillary fronds with mature squamous epithelium and fibrovascular cores, consistent with squamous papilloma. No intraepithelial eosinophils, dysplasia, or malignancy were observed (Figure 2). Gastric biopsies were negative for *Helicobacter pylori*-like organisms.

HPV testing was not performed. Differential considerations included squamous papilloma, early squamous cell carcinoma, verrucous carcinoma, and inflammatory polyps. The patient was advised to continue acid suppression therapy, and a follow-up endoscopy was scheduled in 12 months to monitor for recurrence. She remained asymptomatic at her six-month follow-up. The final diagnosis was solitary squamous papilloma, likely associated with long-standing reflux-related mucosal irritation. 

## Discussion

GERD is a chronic condition caused by the abnormal reflux of gastric contents into the esophagus due to dysfunction of the lower esophageal sphincter (LES), impaired esophageal clearance, delayed gastric emptying, and, in some cases, heightened visceral sensitivity. Key risk factors include obesity, pregnancy, smoking, alcohol intake, consumption of high-fat foods, caffeine, chocolate, certain medications, and connective tissue disorders such as scleroderma [11]. Diagnostic evaluation of GERD includes upper endoscopy to assess mucosal damage, 24-hour pH monitoring (catheter-based or wireless BRAVO system) to quantify acid exposure via the DeMeester score, and impedance-pH monitoring to detect both acid and non-acid reflux episodes [12]. High-resolution manometry is used to evaluate esophageal motility and LES function, particularly to exclude conditions like achalasia [13]. Refractory GERD is defined as the persistence of symptoms despite at least eight weeks of optimized PPI therapy with confirmed adherence and objective evidence of reflux on testing. It requires further workup to rule out functional heartburn, hypersensitivity, or alternative esophageal pathology [13].

This case highlights ESP as a rare but important endoscopic finding in patients with refractory GERD. The observed location in the distal esophagus supports a role for chronic acid exposure in lesion development [1,7]. Chronic mucosal irritation may contribute to a self-perpetuating cycle, wherein GERD promotes the development of ESP, and the presence of ESP exacerbates GERD symptoms, resulting in refractory GERD, as observed in our case [7]. Although direct evidence supporting this cycle is limited, the established association between persistent esophageal irritation and ESP formation lends support to this proposed mechanism. HPV has been detected in up to 87.5% of ESPs in some studies, although its pathogenic role is debated [1,4]. In the absence of papillomatosis or high-risk HPV typing, malignant potential appears very low [2,10]. The typical histological features include mature squamous epithelium overlying a fibrovascular core. Management involves endoscopic excision, which is curative in most cases. Surveillance endoscopy is not always necessary, but may be reasonable in patients with ongoing risk factors or uncertain histology. This case reinforces the diagnostic value of endoscopy in GERD patients with persistent or atypical symptoms and demonstrates the benign nature of ESPs in the setting of chronic mucosal irritation. 

## Conclusions

This case illustrates the relevance of considering ESP in the differential diagnosis of patients with persistent GERD symptoms unresponsive to therapy. The lesion’s location and clinical context suggest a possible link between chronic acid exposure, papilloma formation and symptoms refractoriness. This study highlights a potential link between ESP and refractory GERD, supporting the need for larger retrospective analyses to further investigate this association and explore whether the relationship may represent a self-perpetuating cycle. While the contribution of HPV remains unclear, the benign course and characteristic histology support conservative management following complete excision. Endoscopic evaluation remains essential for diagnosis, treatment and exclusion of malignancy, especially in refractory cases. 
